# A robust sampling technique for realistic distribution simulation in federated learning

**DOI:** 10.1007/s11548-025-03504-z

**Published:** 2025-09-02

**Authors:** Robin Hoepp, Leonhard Rist, Alexander Katzmann, Raghavan Ashok, Andreas Wimmer, Michael Sühling, Andreas Maier

**Affiliations:** 1 Computed Tomography, Siemens Healthineers, Forchheim, Germany; 2 Pattern Recognition Lab, FAU Erlangen-Nürnberg, Erlangen, Germany; 3 Siemens Healthineers, Technology Excellence, Erlangen, Germany

**Keywords:** Federated learning, Distribution shift, Sampling

## Abstract

**Purpose:**

Federated Learning helps training deep learning networks with diverse data from different locations, particularly in restricted clinical settings. However, label distributions overlapping only partially across clients, due to different demographics, may significantly harm the global training, and thus local model performance. Investigating such effects before rolling out large-scale Federated Learning setups requires proper sampling of the expected label distributions.

**Methods:**

We present a sampling algorithm to build data subsets according to desired mean and standard deviations from an initial global distribution. To this end, we incorporate the chi-squared and Gini impurity measures to numerically optimize label distributions for multiple groups in an efficient fashion.

**Results:**

Using a real-world application scenario, we sample train and test groups according to region-specific distributions for 3D camera-based weight and height estimation in a clinical context, comparing a hard data split serving as a baseline with our proposed sampling technique. We train a baseline model on all data for comparison and use Federated Averaging to combine the training of our data subsets, demonstrating a realistic deterioration of 25.3 % on weight and 28.7 % on height estimations by the global model.

**Conclusions:**

Realistically client-biased label distribution can notably harm the training in a federated context. Our sampling algorithm for simulating realistic data distributions opens up an efficient way for prior analysis of this effect. The technique is agnostic to the chosen network architecture and target scenario and can be adapted to any feature or label problem with non-IID subpopulations.

## Introduction

Training artificial neural networks has become state of the art for solving analytical tasks in various domains, including language and speech processing [1] and computer-aided diagnosis in medicine [2]. The main limiting factor for training generalizable neural networks, however, is the availability of sufficient large-scale datasets with satisfactory diversity to cover the relevant problem space. In clinical setups, where the application of neural networks for tasks like image segmentation [3] or classification [4] can greatly accelerate the workflow, this poses the most prominent challenge.

Within the last years, an approach called Federated Learning (FL) has shown growing interest, particularly in contexts in which large datasets are difficult to acquire due to data sensitivity, data privacy or intellectual property, as in the medical domain. Federated Learning is a decentralized training approach that enables the development of neural networks using datasets from multiple sites—such as hospitals or clinical networks—without the need to explicitly share data between them. Instead, FL is applied in a server-client setting, where each client trains a local model using its own data. Afterward, models get merged on the server side to create a global model, which is then redistributed serving as a starting point for further optimization.

However, FL is fundamentally based on the idea of separate datasets with comparable distributions across all contributing parties, i.e., independent and identically distributed (IID), while non- or only partially overlapping data distributions can lead to inhomogeneous local training and impede subsequent model merging. These effects are aggregated and verified in several surveys [5–7]. Countermeasures often focus on specific aspects of client differences, such as annotation quality inconsistencies among clients [8], domain adaptation [9], or statistical imbalances [10]. More general approaches include: FedFusion [11] to find a virtual global distribution or Elastic Weight Consolidation [12] against “catastrophic forgetting” in neural networks.

These methods aim to regulate the influence of different feature distributions of a client on global training to punish counterproductive behavior and to improve generalization. However, the explicit issue of client-biased label distributions (which might roughly be known in advance, e.g., Table 1 for country-specific biases in weight and height) remains not fully solved. In such cases, diverging model gradients might not converge well globally, leading to poor local results. Consequently, clinics might not benefit from a federated training and question its application and effect.

**Table 1:** Comparison of average weight, height, and body mass index (BMI) between men and women across selected countries

Country	Weight [kg]		Height [m]		BMI [kg/$\hbox {m}^2$]	
	Men	Women	Men	Women	Men	Women
Germany	87.8	71.4	1.80	1.66	27.1	25.9
USA	91.5	78.9	1.77	1.63	29.3	29.7
Japan	70.7	55.3	1.72	1.58	24.0	22.1
Nigeria	67.9	62.2	1.70	1.58	23.6	25.0
Brazil	82.5	72.9	1.75	1.62	26.9	27.8

The table highlights variations across different regions. European and American countries typically exhibit higher average values in weight and height compared to Asian and African countries. Data summarized in [13], originally taken from [14]

Investigating such incompatible distributions prior to rolling out FL applications is hence immensely important for industrial players as well as researchers. The FedMedICL framework [15] can benchmark the influence of such distribution shifts with the above-described interpretation but has limitations in simulating realistic datasets by not accounting for complex multidimensional interactions. Control over statistical properties, such as desired means and standard deviations across multiple labels, is not enforced, limiting the realism of the generated subsets. To address this, this work aims for a method to sample subsets from given data with continuous one- or multidimensional labels, enabling the creation of client-specific subsets with predefined statistical properties (e.g., mean and standard deviation). This ensures realistic relationships between attributes, enhancing the validity of FL simulations toward an improvement of the model robustness for real-world medical applications.

For illustration, this work evaluates a typical clinical scenario, using FL for automated patient weight and height estimation using a 3D camera for computed tomography (CT) workflow support. Although weight and height are necessary parameters for patient-tailored contrast administration in CT, they are rarely acquired through measurement, but rather asked, estimated, or omitted, neglecting cost saving potentials and increasing patient risks.^1^ As due to technical reasons a direct integration of a scale into the scanner is not feasible, 3D camera-based estimation is aimed to serve as a simple, noninvasive estimation approach with adequate quality. The concept of an automated weight and height estimation has already been validated in previous research [16, 17], achieving mean errors of 5.6 % for weight and 1.8 % for height estimations on a dataset of about 170,000 depth images taken from more than 1850 individuals. The dataset originated from several cross-national sites and included different diagnostic workflows including patients with weights between 45 and 120 kg and heights between 140 and 200 cm. However, this concept has not been investigated in a FL workflow, further extending the generalization of such a model toward more robustness outside of certain inclusion criteria.

Notably, weight and height distributions show a significant geographical dependence, see Table 1, hindering off-the-shelf Federated Learning and rendering it an excellent exemplary case for our method.

*Contribution* This work provides a tool for realistic data partitioning with known distribution priors given through literature values or pilot studies for realistic FL pre-study quality assessment. A comprehensive pipeline (see Fig. 1) has been developed that enables the simulation of realistic data distribution for FL without the need to collect data from multiple institutions. Our method is based on the chi-squared distance and the Gini impurity measure and uses a greedy sampling scheme. Exemplary, this work investigates the clear demographic shift in a FL scenario for 3D camera-based weight and height estimation. Using two realistically sampled distributions from a single local dataset, we evaluate the training results on both a global and two individually sampled local test sets, mimicking client-specific patient pools, in order to assess the impact of distribution shifts expected in a real-world application.

## Methods

**Fig. 1:**
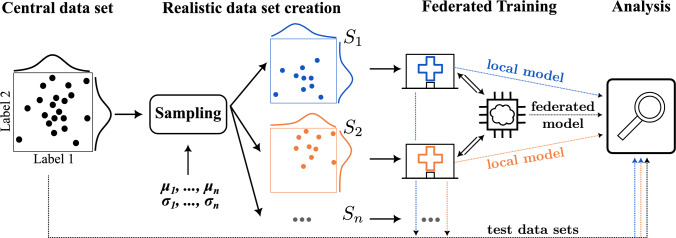
Pipeline of workflow, starting from an initial two-label distribution in black (left)

### Data and architecture

As an exemplary application, we evaluate the feasibility of a FL approach for a 3D camera-based weight and height estimation in a clinical setting, developed for patient-tailored contrast optimization, to increase patient throughput and to reduce human workload [18]. We aim to implement a deep learning-based pipeline to predict patient weight and height prior to the diagnostic procedure, to be used as tuning parameters for specific protocols.

**Fig. 2:**
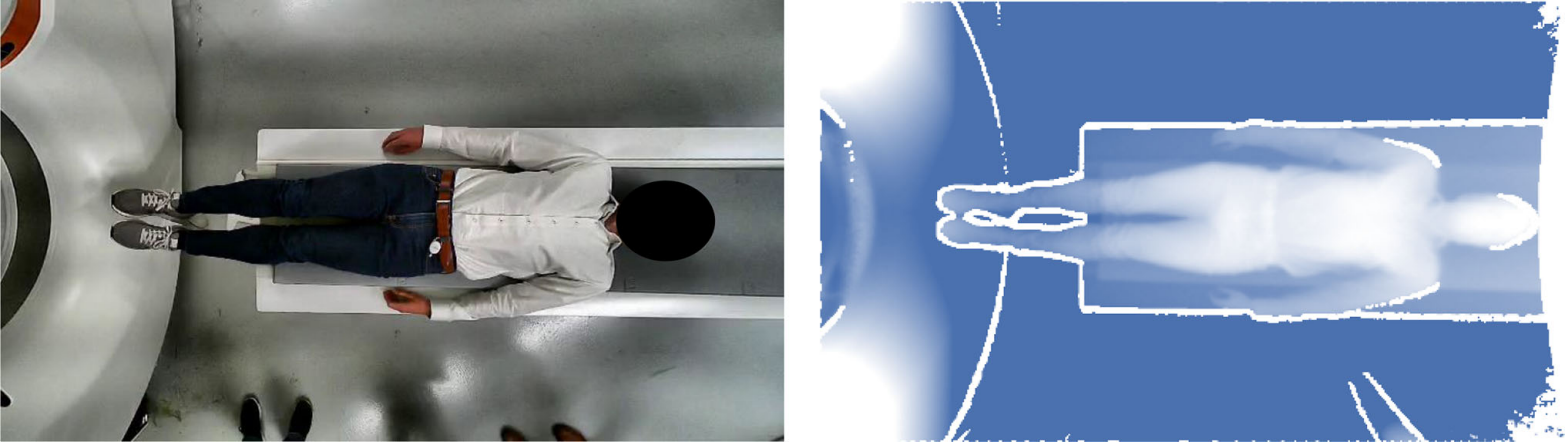
Example images acquired by the 3D Camera. Left: RGB image of a top-down view on the body. Right: Depth image, corresponding to the RGB image. To facilitate visualization, a color mapping scheme has been applied. The depth image has undergone automated post-processing by the camera, aligning it with the corresponding RGB image in terms of field of view and spatial position

The presented approach only utilizes depth images which have been acquired using a time-of-flight camera during patient positioning and discards the corresponding RGB image. The 3D camera for image acquisition is mounted above the scanner table, providing a top-down view of the patient, as shown in Fig. 2.

Similar to Tamersoy et al. [16], images have been transformed into a standardized representation using orthographic re-projection from a virtual camera position centered 2 m above the patient table to account for differences in camera mounting positions. Background artifacts are removed using a threshold of 1.98 m. To account for data privacy, no RGB images have been processed at any time. The dataset consists of 2078 samples, originating from different sites in Europe. Each sample contains a depth image and annotations of patient weight and height, exhibiting mean weights and heights of 79.9 kg ($\sigma$ = 18.7 kg) and 171.6 cm ($\sigma$ = 9.7 cm), respectively.

To predict patient weight and height, we use a ConvNet preceded by a Self-Attention Network [19]. The network architecture is further illustrated in Table 4 (cf. “Appendix”). For decentralized training, we use the Siemens Healthineers Decentralized Training and Federation Platform, based on the NVIDIA Federated Learning Application Runtime Environment (NVFLARE). The averaging method employed is Federated Averaging [20] and the training has been conducted using 50 federation rounds with 20 epochs each.

### Data grouping

To evaluate model performance in FL, the dataset is partitioned in various ways to analyze how specific splits influence training outcome, and whether and in which way biased data will have an influence on the training process. To this end, a comparison split is given through the extreme of a hard data cut by thresholding any of the target variables, i.e., weight or height, at their median. Additional splits that align with an actual real-world scenario are achieved using a sampling algorithm. Therefore, in each experiment, the original dataset is divided into two new distributions that resemble realistic populations, using either a single dimension or multiple dimensions. It must be noted that, given an original distribution of size *N* with mean $\mu$ and variance $\sigma ^2$, any partition with *M* subsets of sizes $n_i$ obeys:

1 $$\begin{aligned} \mu = \frac{1}{N}\sum ^M_i{n_i\cdot \mu _i}, \quad \sigma ^2 = \frac{1}{N}\sum _i^M{ n_i\cdot (\sigma _i^2 + (\mu _i-\mu )^2) }\nonumber \\ \end{aligned}$$

Thus, distributions cannot be arbitrarily derived from any given dataset. Rather, it must already be sufficiently representative for the global distribution, with partitions inherently being only sub-optimally aligned with the desired target distributions, demanding a numerical optimization approach. To this end, it must finally be ensured that the received subsets do not overly deviate from the expected target distributions.

### Sampling scheme

Our approach employs a Monte Carlo-like sampling scheme to iteratively approximate the desired distributions. The algorithm begins by shuffling the dataset. Next, it sequentially assigns each data point to a group guided by the costs associated with the resulting distributional differences between the groups. A cost function *C*(*S*) evaluates how closely the distributions of the subsets $S=\{S_1,...,S_M\}$ align with the target distributions, which are characterized by specified means and standard deviations. By iteratively assigning data points to the group that minimizes the costs, the algorithm generates distributions that closely match the desired target distributions.

The cost function *C* is based on the chi-squared distance $\chi ^2$, a statistical measure that quantifies the difference between an observed distribution *O* (the histogram of the respective group) and an expected distribution *E* (the target distribution). Specifically, for each group, the chi-squared distance is calculated by summing the squared differences between the observed frequencies in the histogram bins and the expected probabilities from the target distribution, normalized by the expected frequencies. For a one-dimensional distribution, the chi-squared distance is given by:

2 $$\begin{aligned} \chi ^2 = \sum _{i=1}^{k} \frac{(O_i - E_i)^2}{E_i} \end{aligned}$$

where $O_i$ represents the observed sample frequency in the $i$-th bin, $E_i$ is the expected frequency in the target distribution, and $k$ is the total number of bins. Note that for any parametric distribution $E_i$ is computable so that $\chi ^2$ only depends on the group assignment. Further, $\chi ^2$ can be computed on any *n*-dimensional joint distribution. Thus, e.g., for a two-dimensional distribution, the chi-squared distance can be extended to the two-dimensional representation of

3 $$\begin{aligned} \chi ^2 = \sum _{i=1}^{k_x} \sum _{j=1}^{k_y} \frac{(O_{ij} - E_{ij})^2}{E_{ij}} \end{aligned}$$

where $O_{ij}$ and $E_{ij}$ represent the observed and expected frequencies in the $ij$-th bin of the 2D histogram, and $k_x$ and $k_y$ are the total number of bins, respectively. To balance the group sizes, the Gini coefficient [21] is incorporated into the calculation, serving as a measure to weight the disparity between the sizes of the two groups. The final cost function *C* for the two groups is defined as

4 $$\begin{aligned} C = \frac{\sum _{i=1}^M\chi ^2_i}{(1 - \text {Gini})^2}\; \quad \text {with}~~\text {Gini} = \frac{\sum _{i=1}^M \sum _{j=1}^M \left| s_i - s_j \right| }{2 \cdot (M - 1) \cdot \sum _{i=1}^M s_i}\qquad \end{aligned}$$

where $\chi ^2_i$ represent the chi-squared distances of the sampled distributions to their respective target distributions, *s* the size of a respective dataset and *M* the number of available datasets. Further, the denominator is squared to emphasize the importance of comparable group sizes. For each experiment, multiple iterations of the algorithm are executed, each initialized with a random permutation of the original dataset. The iteration that yields the lowest costs is preserved. Notably, this sampling can be applied to arbitrary *k*-dimensional distributions and numbers of subsets.

**Fig. 3:**
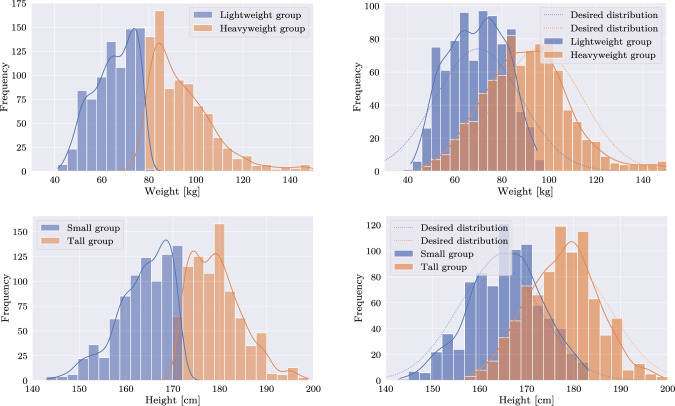
One-dimensional splits of weight and height data. Left: Straightforward split on the median of each label, creating two distinct subsets. Right: Sampling applied to the original dataset, creating two realistic distributions

### Evaluation

For performance assessment, 20 % of the available dataset was held out as a test set, which remained untouched throughout the entire sampling and training process as shown in Fig. 1 (outgoing downward arrow). This subset was randomly selected from the cohort, therefore preserving the original statistical characteristics. During training on each client, a 80/20 train/validation split was applied respectively to the local dataset for the optimization. Figure 1 also illustrates that, for certain experiments, biased test data originating from an individual client were used to analyze the global model’s performance on a client-specific dataset (cf. Appendix).

### Experiments

As a *Central baseline*, a centralized model is trained on the entire train split to serve as a reference to compare the performance of the federated models. Afterward, the following scenarios are investigated:

*Federated Baseline* In a first experiment, this centrally trained model (central baseline) is compared to a federated trained model (federated baseline). In this setup, the data are randomly assigned to one of two clients, generating two datasets with roughly equal statistical properties (IID) without assuming an introduced bias.

*Strict Splits* These initial results are then contrasted with federated networks trained on the aforementioned biased datasets using a strict threshold-based split along the dimension medians.

*Joint Split* In a third experiment, we compare the previous results with a realistic joint distribution split along the weight and height distributions using our proposed sampling technique. We chose target mean values that are equidistant to the original dimension means ($\Delta \approx$ 10 kg/5 cm) and use 30 sampling rounds. In each experiment, we chose two groups for the sake of better visualization and provide the Bhattacharyya distance [22] which allows a comparison from a discretely sampled to continuous target distribution to evaluate the approximation accuracy. A value of 0 indicates perfect overlap, and no overlap goes to infinity.

## Results

### Approximation of target distributions

A strict division into two groups based on a fixed value is trivial, as one group exclusively contains targets greater than a certain threshold, while the other receives the remaining, but may yield unrealistic distributions. In contrast, our proposed sampling can ensure realistic distributions of the data across multiple subsets. Figure 3 depicts a one-dimensional sampling with respect to weight, yielding a lightweight and heavyweight patient group. The Bhattacharyya distances of both groups to their respective target distributions amount to 0.08 and 0.02, respectively. Similarly, 1-d height sampling resulted in comparable subsets, with a shorter (Bhattacharyya = 0.02), and a taller patient group (Bhattacharyya = 0.02), as also shown in Fig. 3.

**Fig. 4:**
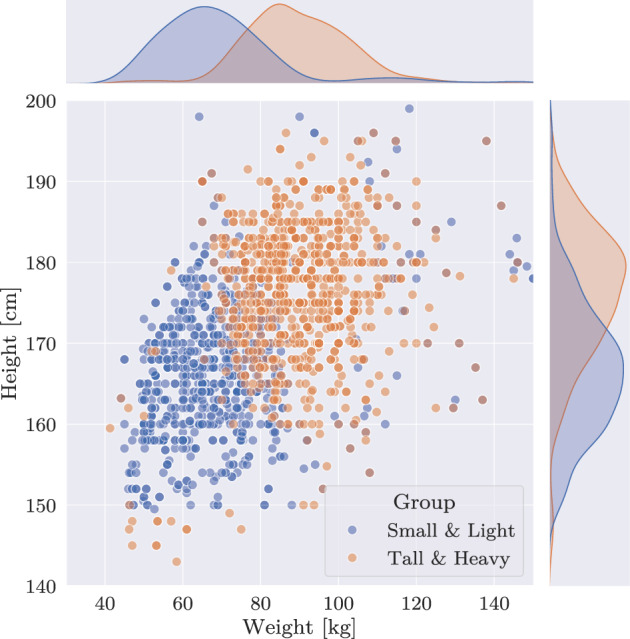
Scatterplot of two subsets, created using the proposed sampling algorithm, separating weight and height jointly. Kernel density estimations illustrate the distribution differences among the subsets

As both only account for realism along a single dimension, a multidimensional sampling on both, weight and height, is required for a realistic data split. To this end, the data were split into a small&lightweight (Bhattacharyya = 0.58) and a tall&heavyweight group (Bhattacharyya = 1.26), see Fig. 4. A comprehensive overview of the desired and achieved statistical properties of each of these distributions is summarized in Table 2.

The runtime of the algorithm depends on the number of iterations, dataset size, and number of clients. It scales linearly with the number of iterations and clients, but exponentially with the number of dimensions, i.e., $\mathcal {O}(n^{d})$, where $n$ is the number of samples and $d$ the number of labels. For instance, 30 iterations with two clients and 2000 samples take about 20 min for one label, but around 110 min for two labels. However, the sampling itself is parallelizable, enabling tremendous runtime reduction on multi-core systems.

**Table 2:**
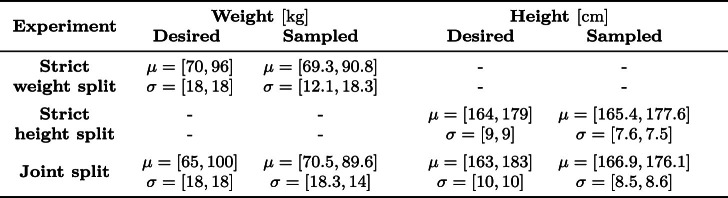
Overview of the statistical properties of the sampled subsets for each experiment, compared to the respective target distributions

### Effect on training

**Fig. 5:**
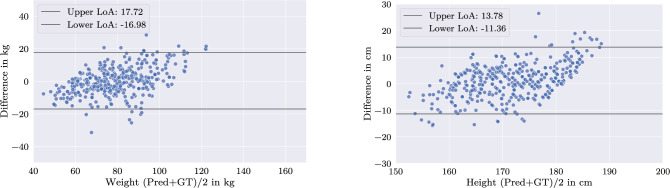
Baseline FL results: Bland–Altman plot of federated model predictions. Weight left, height right. Data points were randomly assigned to one of the FL clients, aligning with the IID data assumption

The performance of the different setups for individual weight and height subgroups is stated in Table 3. The following reported average values are based upon this analysis. Baseline results indicate a comparable performance between a centrally trained and a federated network. The baseline model produces a mean absolute error (MAE) of 6.75 kg for weight and 4.96 cm for height predictions, while the federated model demonstrates a marginal performance enhancement over the centralized approach with a MAE of 6.52 kg for weight and 5.08 cm for height (cf. Fig. 5).

**Fig. 6:**
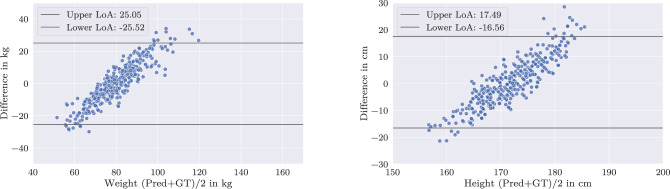
Bland–Altman plot: Federated network, trained on strict splits: lightweight/heavyweight (left) or short/tall (right). Left/right: Predictions of weight/height by the federated model. The strong linearity demonstrates that strict splitting leads to mode collapse in the resulting model

In contrast, strict partitioning of the data into distinct groups considerably impairs the network’s predictive performance for the dimension on which the split is applied. The strong linear trend observed in Fig. 6 highlights the degradation in the network’s ability to predict both labels, with performance on the label used for splitting effectively becoming negligible. This holds equally true for weight- and height-based data splits.

**Fig. 7:**
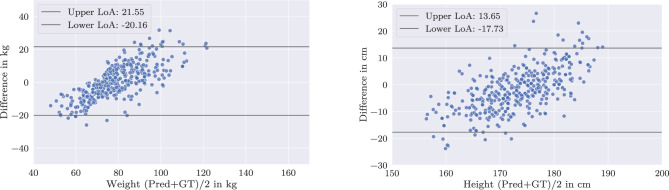
Bland–Altman plot: Federated network, trained the client-biased data splits resulting from the multidimensional proposed sampling. Left/right: Predictions of weight/height by the federated model showing improvements and applicability over a wider range as compared to Fig. 6

On multidimensionally sampled data, the federated network expectedly performs worse than the IID federated baseline. The average MAE increases to 8.17 kg (+ 25.3 %) for weight and 6.54 cm (+ 28.7 %) for height. Particularly for weight, the generalization performance is reduced (cf. Fig. 7). Further tests revealed that, within individual client datasets, the federated model does not provide an advantage over models trained exclusively on the respective client data and may even negatively affect performance, as it is less tailored to the client’s distribution. This is demonstrated in an ablation study in the appendix, where four plots—separately for weight and height—compare the predictive performance of locally trained networks and federated models on a client’s biased dataset. In these plots, the locally trained network achieves lower error on its respective test data compared to the federated model. However, slight improvements are observed in regions underrepresented within the client-specific dataset (cf. “Appendix”).

**Table 3:**
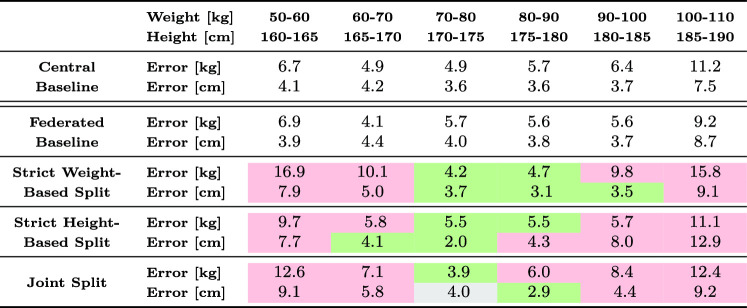
MAE of predictions by differently trained federated networks. Prediction errors for both weight and height increase considerably toward the edges of the distributions, especially for federated models with biased datasets. “Strict” splits refer to experiments, where the data were separated at the median of the respective label. A color mapping scheme compares the respective model to the federated baseline network. For reasons of clarity, the statistics outside the displayed areas are not included in this table. As a result, 130 cases are not listed for weight and 208 cases are not listed for height

In summary, the absence of bias within the clients’ datasets leads to a federated model which achieves comparable accuracy to a centrally trained model that utilizes all available data. However, a general trend of declining prediction performance is observed when biases are introduced into the datasets, i.e., the IID assumption is broken, underlining the need for realistic sampling techniques. Specifically, if the distributions do not overlap due to a strict split, the model’s predictive performance drops drastically. This behavior can be observed in Table 3, where the error for edge cases increases substantially. The improved accuracy around the center or mean of the distribution is attributed to mode collapse in the network, as it predominantly predicts values within this range. In the more realistic two-dimensional resampling scenario, federated training demonstrates slight global improvements, including better performance for edge cases within the respective datasets, albeit with a reduction in predictive accuracy at the core of each distribution.

## Discussion

The results emphasize the importance of addressing client-specific disparities in datasets before finalizing the implementation of a FL approach. In early stages of development, access to sufficiently diverse datasets is often limited, necessitating the partitioning of available data for initial simulations. Simple splits, such as dividing the data into large and small groups, fail to accurately represent real-world scenarios and prevent a meaningful evaluation of federated training. To resolve this, our algorithm generates multiple realistic distributions in a multidimensional space, each with distinct statistical properties, by sampling from an initial pre-development dataset.

When considering algorithmic performance in general, it is important to note that higher performance would be essential in a real clinical setting. The current magnitude and spread of the error are too extensive for reliable use in daily practice, particularly in the presence of edge cases. While more advanced architectures are likely to yield improved results, architectural optimization was not the focus of this work. Instead, the emphasis was placed on developing an early evaluation strategy for such a decentralized training approach. Notably, centralized and IID Federated Learning produced similar outcomes. In contrast, the comparably lower performance of the non-IID federated approaches is explained by the fact that, depending on the individual populations and target distributions, decision criteria may vary significantly across clients, and, consequently, different features are extracted. Thus, aggregation can lead to inconsistencies, hindering effective joint training. Notably, the introduction of biases into individual datasets caused an observable deterioration in training outcomes in all scenarios. However, while hard splits essentially resulted in non-trainability of the network, realistic biases allowed to assess the expected drop in performance both globally and for each distribution individually.

**Fig. 8:**
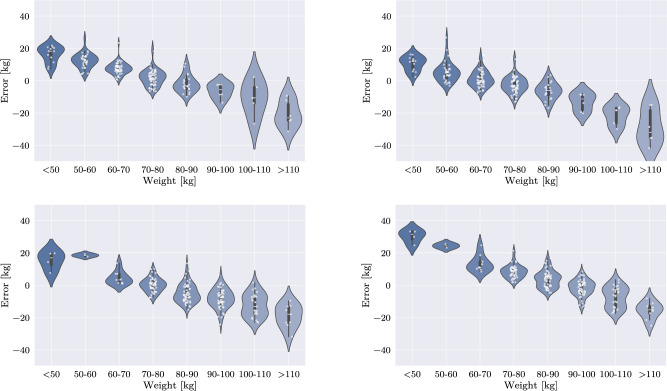
Ablation study for multidimensional sampling on weight and height: Violin plots of federated model predictions versus local model predictions in weight estimation. Top: Comparison on a small/lightweight test data set: Predictions of the federated model (left) versus a local model, trained on a small/lightweight data set (right). Bottom: Comparison on a tall/heavyweight test data set: Predictions of the federated model (left) versus a local model, trained on a tall/heavyweight data set (right). The local model achieves lower error rates in regions with high data coverage within the local distribution compared to the federated model. Conversely, the federated model performs better in regions where the local distribution only contains limited data

**Fig. 9:**
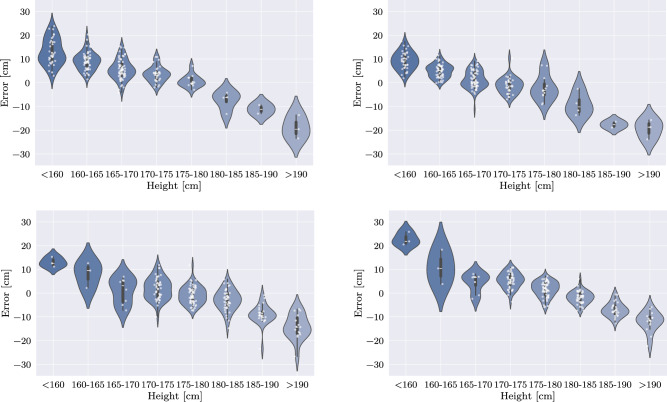
Ablation study for multidimensional sampling on weight and height: Violin plots of federated model predictions versus local model predictions in height estimation. Top: Comparison on a small/lightweight test data set: Predictions of the federated model (left) versus a local model, trained on a small/lightweight data set (right). Bottom: Comparison on a tall/heavyweight test data set: Predictions of the federated model (left) versus a local model, trained on a tall/heavyweight data set (right)

**Table 4:**
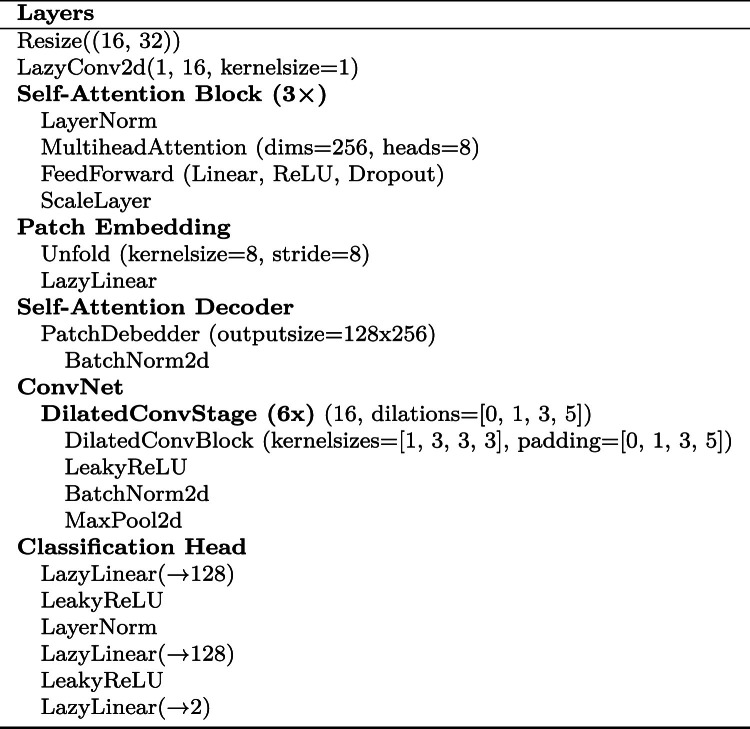
Architecture of the utilized neural network for weight and height estimation

Conducting such an analysis during the initial development phases is crucial to identify and address potential weaknesses in a pipeline and as a tool to research countermeasures. Using the proposed sampling algorithm, it is possible to reliably extract multiple multidimensional subsets from a dataset to examine these issues. This approach enables a FL pipeline to be adapted early on to multicentric, potentially client-biased data, ensuring that the full potential of Federated Learning—namely, accessing an expanded data pool while preserving data privacy—can be effectively realized.

The limitations of our sampling approach are mainly related to the nature of the dataset. To generate multiple sufficiently distinct distributions, the original dataset must exhibit a globally representative data distribution. Further, our method currently uses all available data. While this is essential for smaller datasets, future extensions should consider leaving out data samples if resulting in a globally better approximation.

## Conclusion

This work highlights the harming influence of client-biased label distributions in federated setups and presents a sampling strategy for prior investigation of its effects in a controlled environment. We exemplarily investigate weight and height estimation from 3D depth camera images where global demographic differences can lead to barely overlapping label distributions, complicating the global model training and, thus, decreasing performance compared to locally trained models.

To this end, we present a simple grouping algorithm for splitting a dataset into realistically client-biased subsets for training individual and federated models beforehand. Iteratively minimizing an objective function that includes chi-squared distance for statistical dataset measures and Gini impurity to preserve equal subset sizes, one can approximate given (literature) distributions for federated training before large-scale deployment. We show that such client-biased distributions can have an immense impact on training a federated model, with simple median-based splitting methods leading to no convergence at all, pointing out the need for realistic sampling methods.

This tool aims to boost further countermeasure research and improve on a widely-present issue in non-IID Federated Learning. In scenarios with initially limited access to data, the presented sampling scheme can be used to synthesize client-specific non-IID data, opening up several directions for future work. First, the algorithm could be extended to support a wider range of target distributions beyond the Gaussian distribution to enable adaptation to all real-world scenarios. Second, comparisons with implemented non-simulated scenarios would further validate the reliability of our simulation outcomes. Third, although the current work focuses on regression tasks, the approach should also be evaluated for other problem types, such as classification and segmentation of volumetric data, to assess its generalizability across application domains.

## Appendix

See Figs. 8, 9 and Table 4.

## Footnotes

1 Contrast agent underdosage in CT may lead to images lacking diagnostic quality. Overdosage, on the other hand, puts unnecessary strain on the patient’s body and is costly for the hospital.
